# Suicide Around the Anniversary of a Parent’s Death in Sweden

**DOI:** 10.1001/jamanetworkopen.2023.6951

**Published:** 2023-04-11

**Authors:** Alessandra Grotta, Can Liu, Ayako Hiyoshi, Lisa Berg, Ichiro Kawachi, Jan Saarela, Mikael Rostila

**Affiliations:** 1 Department of Public Health Sciences, Stockholm University, Stockholm, Sweden; 2 Centre for Health Equity Studies, Stockholm University/Karolinska Institutet, Stockholm, Sweden; 3 Clinical Epidemiology Division, Department of Medicine Solna, Karolinska Institutet, Stockholm, Sweden; 4 Clinical Epidemiology and Biostatistics, School of Medical Sciences, Örebro University, Örebro, Sweden; 5 Department of Social and Behavioral Sciences, Harvard T. H. Chan School of Public Health, Boston, Massachusetts; 6 Demography Unit, Åbo Akademi University, Vaasa, Finland

## Abstract

**Importance:**

Bereavement following parental death experienced in adulthood may be associated with suicide over many years, but this risk has received scant attention.

**Objective:**

To investigate whether the risk of suicide increases among adult children around the anniversary of a parent’s death.

**Design, Setting, and Participants:**

This case-crossover study used Swedish register-based longitudinal data from 1990 to 2016, based on the entire national population. Participants included all adults aged 18 to 65 years who experienced parental death and subsequently died by suicide. Conditional logistic regression was used to quantify the association between the anniversary (or preanniversary and postanniversary periods) and suicide, controlling for time-invariant confounding. All analyses were stratified by sex of the offspring. The analyses were also stratified by the sex of the deceased parent, time since parental death, age, and marital status. Data analyses were performed in June 2022.

**Exposures:**

Anniversary of a parent’s death (or preanniversary and postanniversary periods).

**Main Outcomes and Measures:**

Suicide.

**Results:**

Of 7694 individuals who died by suicide (76% intentional self-harm), 2255 (29%) were women, and the median (IQR) age at suicide was 55 (47-62) years. There was evidence of an anniversary reaction among women, with a 67% increase in the odds of suicide when exposed to the period from the anniversary to 2 days after the anniversary, compared with when not being exposed (odds ratio [OR], 1.67; 95% CI, 1.07-2.62). The risk was particularly pronounced among maternally bereaved women (OR, 2.29; 95% CI, 1.20-4.40) and women who were never married (OR, 2.08; 95% CI, 0.99-4.37), although the latter was not statistically significant. An increased risk of suicide from the day before up to the anniversary was observed among women bereaved between the ages of 18 and 34 years (OR, 3.46; 95% CI, 1.14-10.56) and between the ages of 50 and 65 years (OR, 2.53; 95% CI, 1.04-6.15). Men had an attenuated suicide risk for the period from the day before up to the anniversary (OR, 0.57; 95% CI, 0.36-0.92).

**Conclusions and Relevance:**

These findings suggest that the anniversary of a parent’s death is associated with an increased suicide risk among women. Women bereaved at younger or older ages, those who were maternally bereaved, and those who never married appeared to be particularly vulnerable. Families and social and health care professionals need to consider anniversary reactions in suicide prevention.

## Key Points

**Question:**

Does the risk of suicide among adult children increase around the anniversary of a parent’s death?

**Findings:**

In this case-crossover study of 7694 individuals who died by suicide, an increased suicide risk was found for women around the anniversary of a parent’s death.

**Meaning:**

These findings suggest that families and social and health care professionals need to consider anniversary reactions in suicide prevention among adults who have lost a parent, especially bereaved women.

## Teaser

This case-crossover study investigates whether the risk of suicide increases among adult children around the anniversary of a parent’s death.

## Introduction

Most people lose a parent during adulthood. Such bereavement could nevertheless lead to long-lasting grief-related physical and mental health problems,^1,2,3,4,5,6,7,8^ including an increased risk of depression, suicidal thoughts, and psychiatric problems.^1,2,3^ It is also thought to be associated with an increased risk of suicide, although the findings have been inconsistent.^4,6,9^

Whether previously found associations between parental death and mental health outcomes reflect the impact of bereavement per se is not clear, because associations may be confounded by genetic vulnerability or shared social conditions among both parents and offspring. One methodological approach to test the causal impact of bereavement is to investigate how mental health disorders precipitate around anniversaries, a phenomenon known as anniversary reactions.^10,11^ Anniversaries may trigger grief reactions and contribute to an acute deterioration of mental health and, thereby, an increased risk of suicide. At the same time, anniversaries are exogenous to shared intergenerational characteristics. An increased risk of adverse outcomes around the anniversary may, therefore, provide an indication of a causal effect of bereavement.

Suicide around the anniversary of a parent’s death has generally received scant attention in the bereavement literature. To date, anniversary reactions have been mainly investigated among adults who lost a spouse or a child,^12,13^ and only 2 studies have been conducted on the anniversary of parental death. One of them, a Swedish study on children and youth bereaved before the age of 25 years,^5^ has shown that girls were more susceptible to suicide-related behavior around anniversaries than boys, and that also the anticipation of the anniversary increased the risk of mental health disorder, consistent with previous research on anniversary reactions.^14^ Another study,^15^ based on a small sample, found an increased risk of suicide around the anniversary of paternal, but not maternal, death. Although previous studies^7,8,16,17^ have found that parental death experienced at younger ages may have long-lasting outcomes on mental health, to our knowledge, no study has investigated grief reactions around anniversaries by offspring’s age at the time of loss. Furthermore, individuals who lose a parent in young adulthood are less likely to have started their own family, whose support could buffer against acute grief reactions.^18^ Thus, marital status may affect the anniversary reaction.

According to our previous research,^12^ we hypothesize that the date of parent’s death may be a stronger trigger than, for example, the date of a parent’s birth. Therefore, we focused on the risk of suicide around the date of a parent’s death in this study. Using Swedish nationwide register-based data, we designed a case-crossover study to investigate whether the risk of suicide increases around the anniversary of parental death in adult men and women. By design, the case-crossover study controls for all observed and unobserved time-invariant confounders, because it takes advantage of within-individual comparisons. We hypothesized that the anniversary reaction decreases over time and differs by the sex of the parent and the age and marital status of the offspring.

## Methods

### Study Population

For this case-crossover study, we used Swedish multiregister linked data of all individuals registered any time between 1990 and 2016 in the Longitudinal Integrated Database for Health Insurance and Labour Market Studies, a data set composed of annually compiled socioeconomic information for all Swedish residents aged 16 years and older.^19^ This total population consisted of 13 030 439 individuals; the study enrollment flowchart is shown in eFigure 1 in Supplement 1. We first excluded 4 335 522 individuals for whom no biological parents could be found, who primarily consisted of people born abroad, people born before 1932, and people who lost both parents before 1990. Individuals who were adopted were additionally excluded (67 560 individuals). After linkages to the Cause of Death Register,^20^ we excluded 40 535 individuals with an incomplete date for own or parental death and 5 762 336 individuals who did not experience a parental death in 1991 to 2015. Linkages to the Total Population Register^21^ allowed us to further exclude 159 636 individuals who had lived abroad or whose parents had lived abroad. Thereafter, we excluded 165 698 individuals who experienced the first parental death occurring in 1991 to 2015 before age 18 years or after age 65 years. Since only cases with events (suicides) can contribute to the case-crossover study design, we finally restricted our sample to individuals who died by suicide. These were defined according to the *International Classification of Diseases*, as having the *International Statistical Classification of Diseases and Related Health Problems, Tenth Revision* codes X60 to X84 (intentional self-harm) or Y10 to Y34 (event of undetermined intent), or the *International Classification of Diseases, Ninth Revision *codes E950 to E959 (intentional self-harm) or E980 to E989 (event of undetermined intent), as the main or secondary cause of death. Register data were collected until December 31, 2016. The Swedish Ethical Review Authority approved this study. Informed consent from the participants was not necessary because it is not required for register-based research in Sweden. The study followed the Strengthening the Reporting of Observational Studies in Epidemiology (STROBE) reporting guidelines.

### Study Design

We designed a time-stratified, case-crossover study.^22,23^ The case-crossover design was developed to investigate the effect of transient exposures on the risk of acute events.^22^ It is characterized by the fact that only cases are selected, and no control group is needed since each individual is observed at different times and serves as their own control. In this study, we implemented a time-stratified version of the design, where the suicide day is the case day and control days are defined as the same days of the week within the same month in which the suicide occurred.^23^ For example, if an individual died by suicide on Monday, March 8, 2010, this day is set to be the case day, whereas control days were set to the other Mondays of the same month of the same year, that is, March 1, 15, 22, and 29, 2010 (eFigure 2 in Supplement 1). Thus, for each case day there are 3 or 4 control days. Since each of the individuals acts as their own control, time-invariant confounders are controlled for (eg, sex-specific or genetic predisposition to suicide). Furthermore, by choosing control days as the same weekdays within the same month as for the case day, time-variant confounders due to day of the week or seasonality are controlled for.^23,24^

### Exposures

In accordance with the study by Stickley et al,^23^ we created a set of 29 dummy exposure variables indicating whether the case day (the day of offspring’s suicide) or its control days fell in specified time periods, which ranged between 0 and 14 days before and 0 to 14 days after the anniversary of a parental death. The exposure variable used to assess the risk of suicide on the anniversary was assigned a value equal to 1 if the case (or the control) day coincided with the anniversary, 0 otherwise. The exposure variable used to assess the risk of suicide over a 2-day period including the anniversary and the day before the anniversary was assigned a value equal to 1 if the case (or the control) day fell in this period, 0 otherwise. In the same way, we created exposure variables indicating whether the case or control day fell in the time period ranging from the anniversary day up to 14 days before the anniversary day. Analogously, we created 14 exposure variables for postanniversary periods. In eFigure 3 in Supplement 1, we illustrate the periods targeted by each exposure variable, and in eFigure 4 in Supplement 1, we summarize how data were set for the analysis.

### Potential Effect Modifiers

We investigated the role of the sex of the deceased parent (mother or father), time of suicide since parental death (0-5, 0-10, or 0-20 years; in comparison to the main analysis concerned with the whole follow-up period), marital status at the time of suicide (ever married or never married), and age at the time of loss (young, 18-34 years; early middle age, 35-49 years; or late middle age, 50-65 years). Cutoff points for age were chosen according to previous literature.^25^

### Statistical Analysis

To investigate whether the risk of suicide increases around anniversaries, we used conditional logistic regression models to estimate odds ratios (ORs) with 95% CIs. We ran 29 models, 1 for each of the exposure variables. As previously explained, exposure variable indicates whether the case (or control) day coincided with the anniversary or fell within a time frame from the anniversary day up to 14 days before or after the anniversary day. Therefore, the OR can be interpreted as the ratio of the odds of the risk of suicide when an individual is exposed to the anniversary of parental death (or to a specified preanniversary or postanniversary period) and the odds of the risk of suicide when the individual is not exposed. SEs accounted for the clustered structure of the data. All analyses were stratified by the sex of the bereaved individuals. We further stratified the analyses by the sex of the deceased parent, time of suicide since parental death, marital status at suicide, and age at the time of the loss.

For the main (nonstratified) analysis, we also estimated the average semielasticities, that is, the average percentage change in the risk of suicide when the individual is exposed compared with when the individual is not exposed.^26,27^ To assess robustness of our main results to potential bias arising from within-subject exposure dependency,^28^ we randomly selected 1 control day among the available control days, repeated the sampling 100 times, and plotted the average OR with the 2.5th and 97.5th percentiles.

Two-sided *P* < .05 was considered statistically significant. All analyses were performed in June 2022 using Stata statistical software version MP 15.1 (StataCorp).

## Results

In our population of individuals who lost a parent in 1991 to 2015, 7694 individuals died by suicide (76% by intentional self-harm, 24% by undetermined intent), and 2255 (29%) of them were women. The median (IQR) age at suicide was 55 (47-62) years, and the median (IQR) time occurring between parental death and suicide was 7 (3-12) years. Descriptive characteristics are presented in the Table. Of 7694 suicides, we observed 16 (0.21%) on anniversary dates (8 [0.15%] for men and 8 [0.35%] for women) and 567 (7.37%) during the 2-week period preceding or following the anniversary (398 men [7.32%] and 169 women [7.49%]) (eTable in Supplement 1).

**Table.** Characteristics of Individuals Aged 18 to 65 Years Who Experienced Parental Death Between 1991 and 2015 and Who Subsequently Died by Suicide

Characteristic	Individuals, No. (%)		
	Women (n = 2255)	Men (n = 5439)	Total (N = 7694)
Age at suicide, median (IQR), y	55 (48-62)	54 (47-62)	55 (47-62)
Time between parental death and suicide, median (IQR), y	7 (3-12)	7 (3-12)	7 (3-12)
Parental death			
Mother	1070 (47.5)	2575 (47.3)	3645 (47.4)
Father	1185 (52.5)	2859 (52.6)	4044 (52.6)
Mother and father (same date)	0	5 (0.1)	5 (0.1)
Age at first parental death			
Young (18-34 y)	309 (13.7)	846 (15.6)	1155 (15.0)
Early middle age (35-49 y)	1018 (45.1)	2486 (45.7)	3504 (45.5)
Late middle age (50-65 y)	928 (41.2)	2107 (38.7)	3035 (39.5)
Marital status at suicide			
Never married^a^	691 (30.6)	2341 (43.0)	3032 (39.4)
Ever married	1560 (69.2)	3080 (56.6)	4640 (60.3)
Missing^b^	4 (0.2)	18 (0.3)	22 (0.3)

^a^ Marriage includes registered partnership.

^b^ Missing observations were not included in the analyses stratified by marital status at suicide.

**Figure 1.**
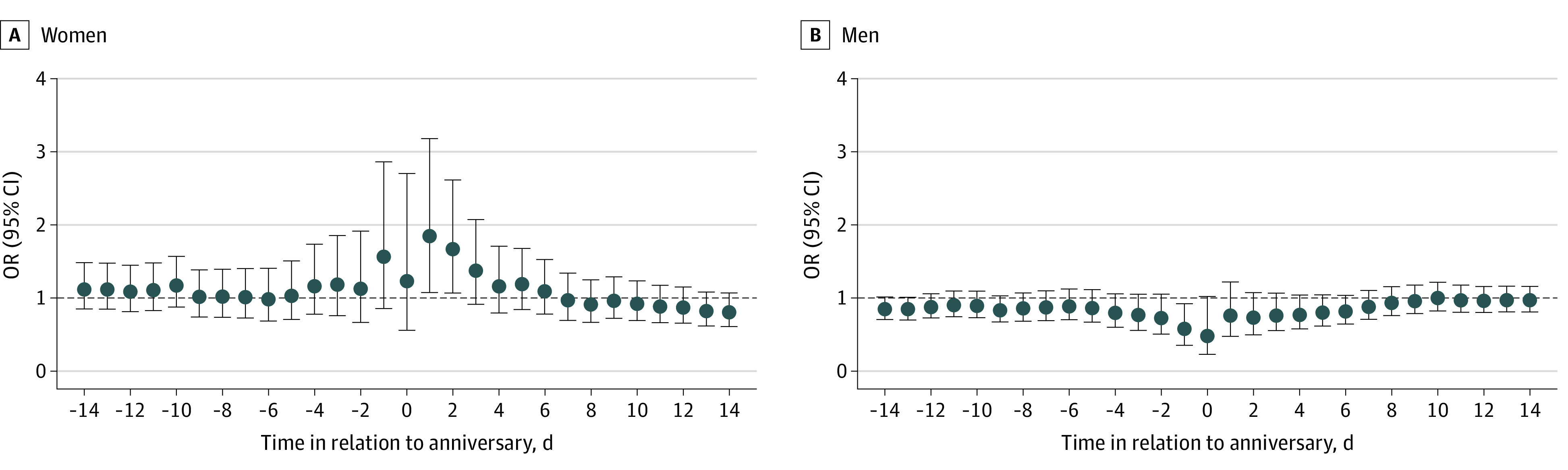
Association Between the Anniversary of a Parent’s Death and Suicide, Stratified by Sex Graphs show odds ratios (ORs) and 95% CIs for the association between anniversary (or preanniversary and postanniversary periods) and suicide among women (A) and men (B).

We found a statistically significant increase in the risk of suicide among women in the period ranging from the anniversary day to 2 days after (OR, 1.67; 95% CI, 1.07-2.62) (Figure 1A). The risk around the anniversary was more pronounced over the first 5 years following parental death (eFigure 5 in Supplement 1). Among men, we found an attenuated risk for the period from the day before up to the anniversary (OR, 0.57; 95% CI, 0.36-0.92) (Figure 1B) and similar patterns over time (eFigure 5 in Supplement 1).

**Figure 2.**
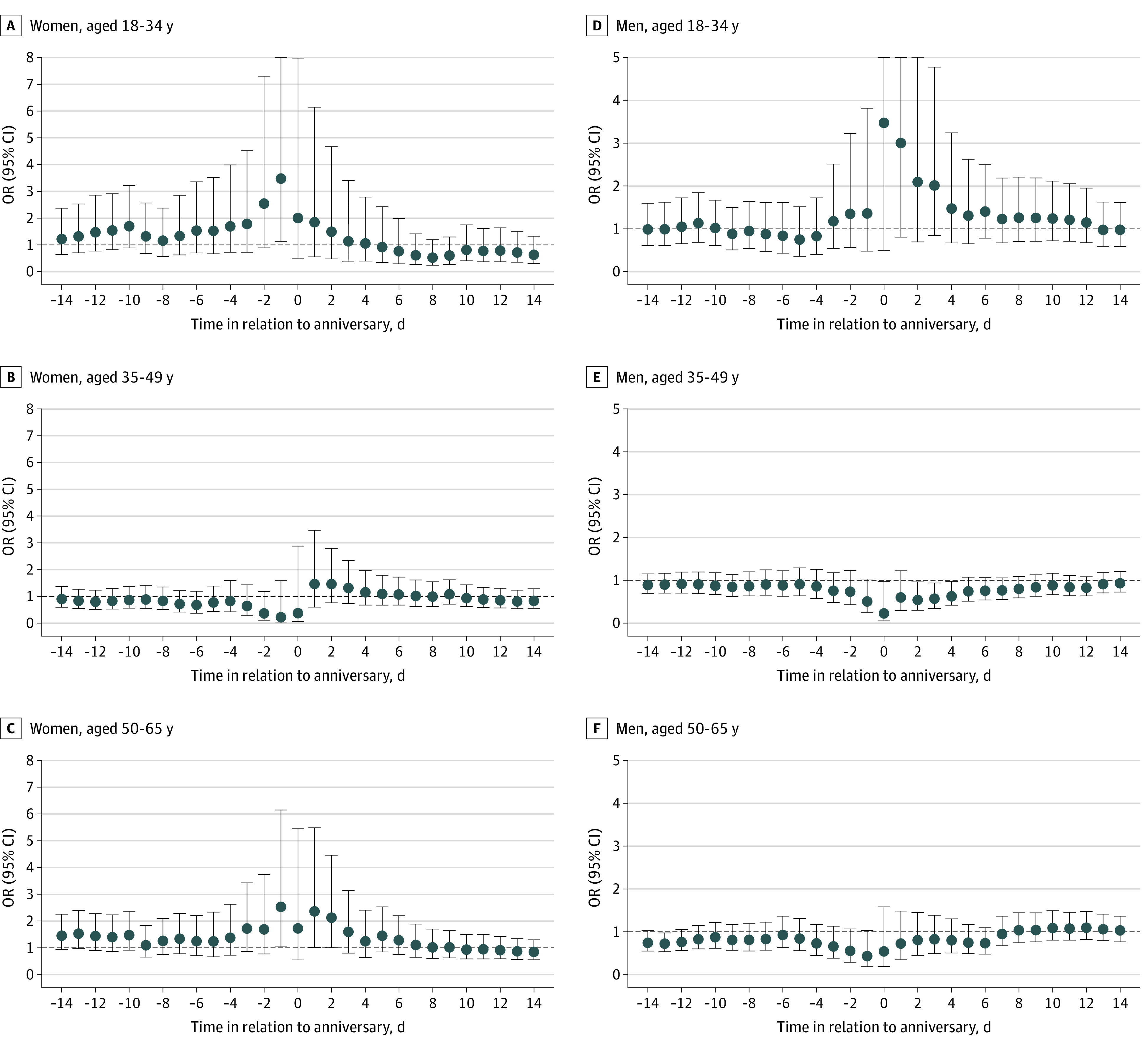
Association Between the Anniversary of a Parent’s Death and Suicide, Stratified by Age at Parental Death Graphs show odds ratios (ORs) and 95% CIs for the association between anniversary (or preanniversary and postanniversary periods) and suicide among women and men bereaved at age 18 to 34 years (A and D), 35 to 49 years (B and E), and 50 to 65 years (C and F). Upper 95% CIs were truncated at 8 in panel A and at 5 in panel D.

**Figure 3.**
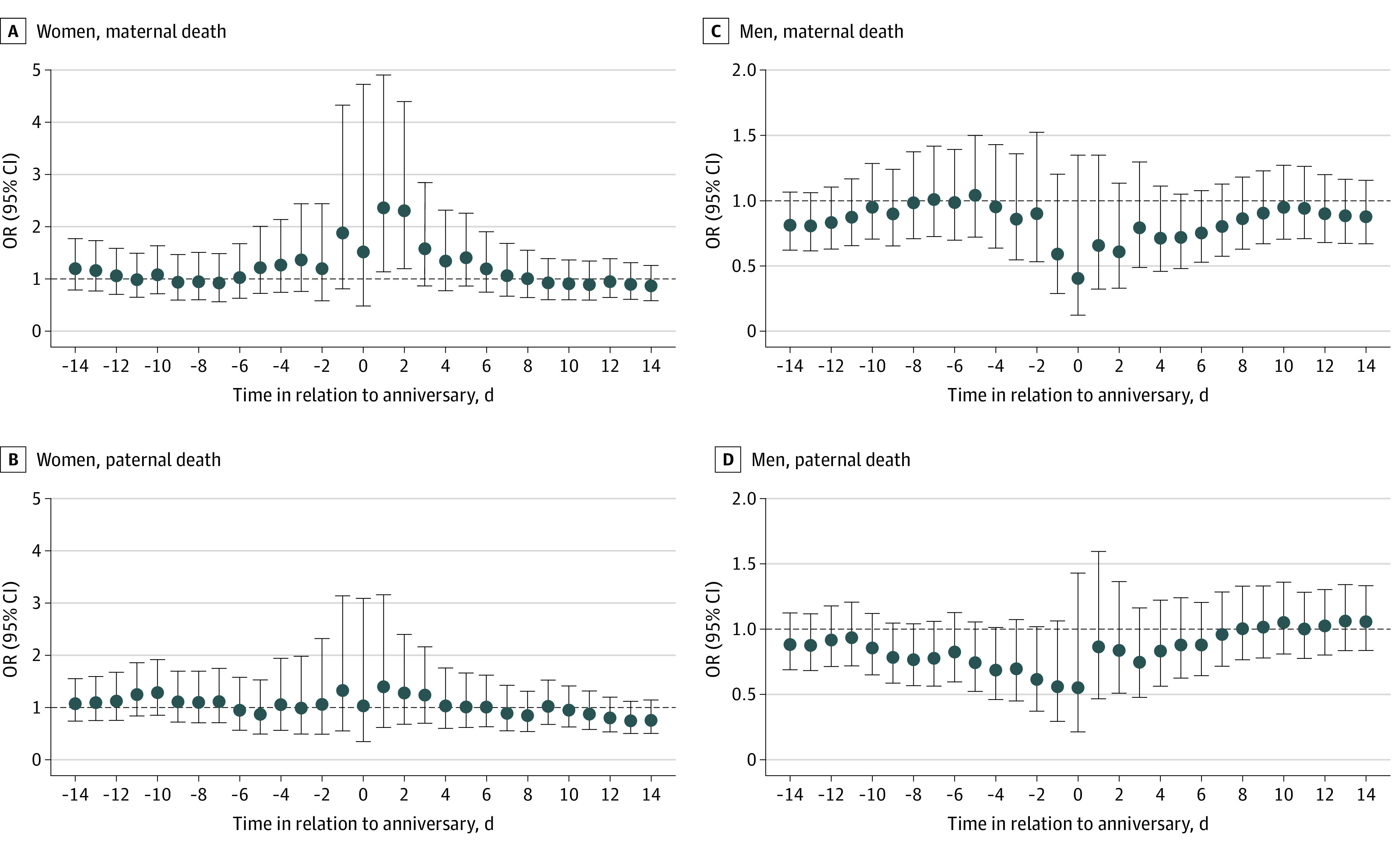
Association Between the Anniversary of a Parent’s Death and Suicide, Stratified by Maternal and Paternal Death Graphs show odds ratios (ORs) and 95% CIs for the association between anniversary (or preanniversary and postanniversary periods) and suicide among women and men following maternal death (A and C) and paternal death (B and D).

**Figure 4.**
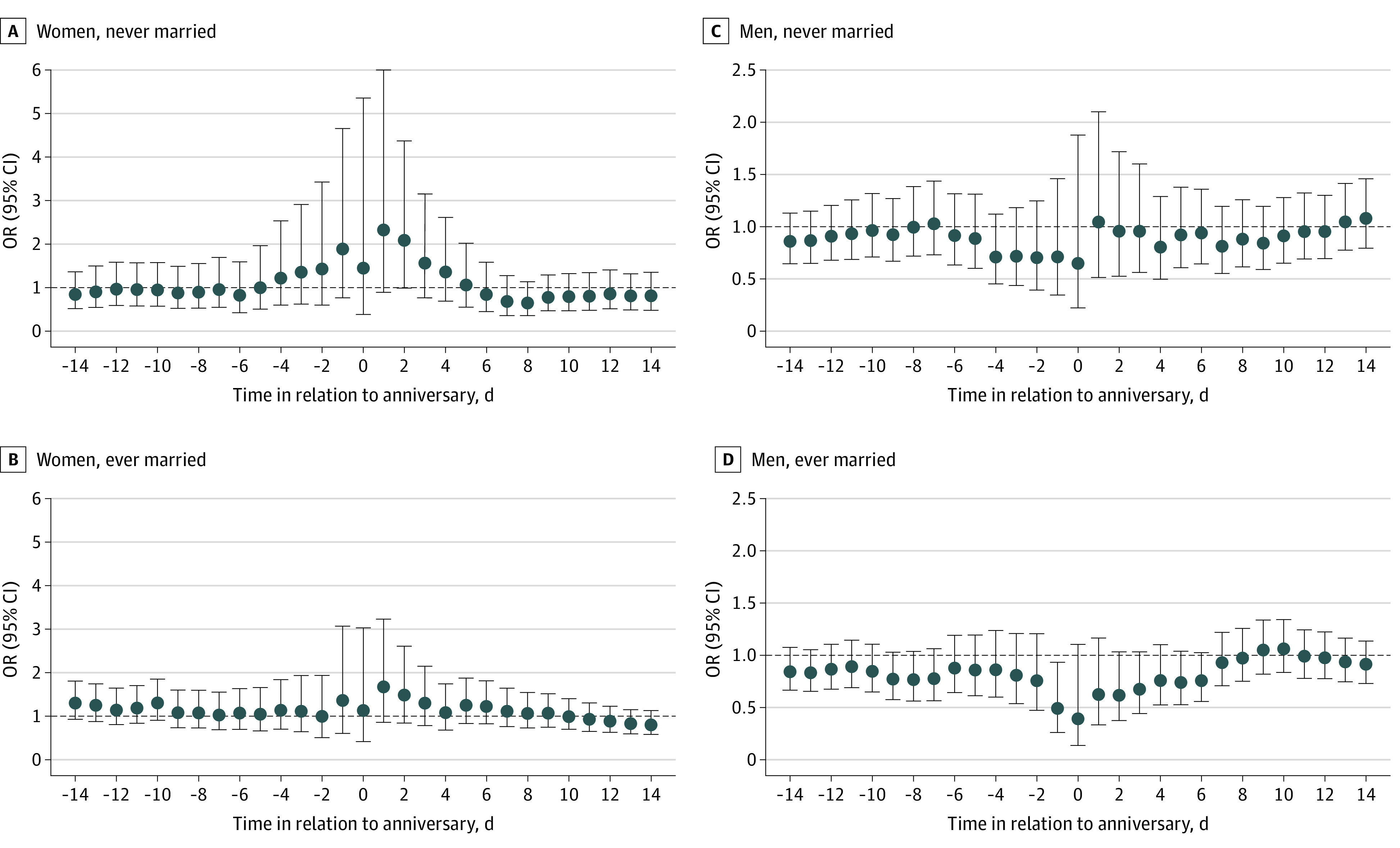
Association Between the Anniversary of a Parent’s Death and Suicide Among Women and Men, Stratified by Marital Status Graphs show odds ratios (ORs) and 95% CIs for the association between anniversary (or preanniversary and postanniversary periods) and suicide among women and men never married (A and C) and ever married (B and D) at the time of suicide.

We found a significant increase in the risk from the day before up to the anniversary for women losing a parent at ages 18 to 34 years (OR, 3.46; 95% CI, 1.14-10.56) and ages 50 to 65 years (OR, 2.53; 95% CI, 1.04-6.15). Women bereaved at ages 35 to 49 years did not have a significant increase in the risk of suicide around the anniversary (Figure 2). Among men bereaved at ages 18 to 34 years, there was an increased risk on the anniversary but it was not significant (OR, 3.46; 95% CI, 0.48-25.00) (Figure 2). A significant increased risk was observed among maternally bereaved (OR, 2.29; 95% CI, 1.20-4.40 for the period from the anniversary to 2 days after), but not paternally bereaved, women (Figure 3). Among men, no clear patterns were observed regarding maternal or paternal death (Figure 3). The increase in the risk was smaller among ever married women (OR, 1.48; 95% CI, 0.84-2.61) compared with never married women (OR, 2.08; 95% CI, 0.99-4.37), although both were not statistically significant, for the period from the anniversary to 2 days after (Figure 4). A slight decrease in the risk of suicide around the anniversary was observed among ever married men (Figure 4).

The analysis estimating semielasticities showed an average additional increase in the risk of suicide among women for the period ranging from the anniversary to the day after (semielasticity, 0.48; 95% CI, 0.06 to 0.89) and a decrease among men for the period from the day before the anniversary up to the anniversary (semielasticity, −0.43; 95% CI, −0.80 to −0.06) (eFigure 6 in Supplement 1). The sensitivity analysis showed patterns consistent with the main findings (eFigure 7 in Supplement 1).

## Discussion

In this case-crossover study using Swedish national register data, we found evidence of an anniversary reaction among women, with an increased risk of suicide most consistently observed during the 2-day period following the anniversary of a parent’s death. Among men, we observed a reduced risk of suicide around the anniversary.

Only a few studies^5,13,15^ have investigated anniversary reactions among people who lost a loved one, and the majority of the research has used small or convenience samples. To our knowledge, this is the first study based on the total working age population that investigates how the anniversary reaction varies among adult children according to time since parental death, marital status, and age at the time of the parent’s death. Moreover, previous studies have often investigated how the overall risk change within a 1-month period around the anniversary, without differentiating between preanniversary or postanniversary increases in the risk.^13,15^ Using a case-crossover design on a large national sample, our study was able to investigate periods that were associated with a higher risk of suicide before and after the anniversary, in line with a previous study focusing on younger individuals.^5^

Although the loss of a parent during childhood has received attention in previous research, bereavement following the loss of a parent experienced during adulthood has been largely overlooked. Even though losing a parent during adulthood is an expected event, it does not necessarily imply that the offspring’s psychological and physical health will be unaffected.^29,30^ Our findings of an increased risk of suicide in women around the anniversary provides an indication that bereavement may be associated with mental health outcomes among adult offspring. The claim for an association is made stronger by the use of the anniversary as an exposure exogenous to shared intergenerational characteristics, as well as the adoption of a within-individual design.

Consistent with previous studies,^5,12,15^ our findings showed clear differences in anniversary reactions between men and women. Our results suggest that anniversaries may trigger grief among women, but we did not find any evidence of an anniversary reaction among men, but rather a decreased risk of suicide. Analogous differences between men and women have been observed in the mortality risk among parents who lost a child^12^ and in suicide-related behavior among children and youths who lost a parent.^5^ We could speculate that women have stronger relationships with their parents, potentially relating to caregiver roles that women assume more often than men.^31^ It is worth noting that a slight decrease of the risk on the anniversary was observed among women, too, potentially reflecting that both men and women engage in rituals on the anniversary, thus being less likely to complete suicide on that day. Even if less likely, it could also be speculated that men receive more support from people around them near the anniversary. This hypothesis would be consistent with our findings showing a decrease of suicide only in ever married men. However, the reasons for differences by sex, in particular the decreased risk of suicide among men, deserve further investigation.

We observed an anniversary reaction among women who lost a parent either before age 35 years or after age 50 years, with the greatest association being found in the youngest group. The highest risk observed among women losing a parent while being young is in line with previous literature,^16,17^ showing that parental death experienced during young adulthood may be particularly detrimental, because perceived parental support is still high while transitioning to adulthood.^29^

No association was found among women losing a parent between ages 35 and 50 years, which may reflect the protective role of a large and diverse social network that women tend to have at these ages, by taking on several roles within the family and at work.^32^ This explanation is also consistent with another finding of this study, showing ever married women to be less vulnerable to the anniversary reaction, again reflecting the protective role of having their own family. Correspondingly, loosened social connections, along with retirement or adult children leaving home, may underlie the association seen among women losing a parent in late adulthood.

When stratifying analysis by the time since loss, we found that the anniversary reaction was more pronounced during the first 5 years. Consistent with our findings, a study^33^ conducted in the US, including 768 widowers, found that the intensity of anniversary reactions decreased quickly during the first few years after bereavement. The aforementioned Swedish study^5^ also found that the anniversary reaction following the death of a parent was more pronounced during the first few years.

Finally, we investigated the anniversary of mother’s and father’s death separately for men and women. Results suggest that, among women, maternal deaths were potentially a stronger trigger for anniversary reactions compared with paternal deaths. This result is aligned with findings from previous empirical studies, which have described women’s worse outcomes following the death of a mother compared with the death of a father.^17,18,34^ These findings are consistent with the unique closeness characterizing the mother-daughter ties.^35^ No differences between maternal and paternal death were observed among men.

### Strengths and Limitations

Our study has several strengths. First, to the best of our knowledge, this is the first large-scale study using data from highly complete national registers with minimal loss to follow-up to investigate suicides around the anniversary of a parent’s death experienced during adulthood. By doing so, we have contributed to the literature on bereavement during adulthood, which has often focused on the death of a child or a spouse. Second, we used within-individual comparisons, which allowed us to control for potential bias due to time-invariant confounding. Moreover, control days were set to be the same weekdays within the same month as the suicide event, thus controlling for time-variant confounders due to weekday and seasonality.

This study also has some limitations. First, there can be potential misclassification of suicide cases, given that our definition of suicide includes events of undetermined intent, some of which may have been accidents. If accidents were more likely to be identified as suicide if occurred close to the anniversary, we may have overestimated the true association. However, since suicides are more likely to be misclassified as accidents than the other way around, we can assume that, if anything, we presumably underestimated the true association. Second, there can be unmeasured time-varying confounding, although we adjusted for weekday and seasonal confounding. Third, even though the whole population was used, the study population size was limited, which resulted in wide confidence intervals for some of the stratified analyses. Because of the limited sample, it was also not possible to stratify by different causes of parental death. Fourth, our sample is not fully representative of the general population, given that the population born outside Sweden whose parents were not registered in Sweden and individuals having lived abroad were excluded. Fifth, we did not investigate anniversary reactions around other important dates, such as birthdays of the deceased person.^12^ Sixth, although we performed several tests, our study did not adjust for multiple testing. On that account, the strength of the evidence presented may be weaker than implied by our analyses, and future confirmatory studies would be useful.

## Conclusions

In conclusion, the anniversary of a parent’s death was associated with an increased risk of suicide. Women bereaved at younger or older ages, those who were maternally bereaved, and those who were never married appeared to be particularly vulnerable. Families and social and health care professionals need to consider such anniversary reactions, especially for bereaved women. Practitioners within mental health services may routinely record death anniversaries, detect signs of adverse mental health during anniversaries, and facilitate timely access to targeted support.

## Notes

Supplement 1. **eTable.** Suicides Around the Anniversary of a Parent’s Death Among Individuals Aged 18-65 Who Experienced Parental Death Between 1991 and 2015 (n=7,694) **eFigure 1.** Flowchart of the Study Population **eFigure 2.** Example of Selection of Control Days, for Case Day on Monday, March 8, 2010 **eFigure 3.** Target Periods for Each Exposure Variable **eFigure 4.** Illustration of How the Dataset and Variables Were Created for the Case-Crossover Study **eFigure 5. **Association Between the Anniversary of a Parent’s Death and Suicide Among Women and Men, Stratified by Time Since Parental Death **eFigure 6. **Association Between the Anniversary of a Parent’s Death and Suicide Among Women and Men Using Semielasticities **eFigure 7.** Association Between the Anniversary of a Parent’s Death and Suicide Among Women and Men (Sensitivity Analysis After Randomly Selecting 1 Control Day)

Supplement 2.
Data Sharing Statement
