# Supplementary material for: Suicide Around the Anniversary of a Parent’s Death in Sweden
Source: JAMA Netw Open. 2023 Apr 11;6(4):e236951. doi: 10.1001/jamanetworkopen.2023.6951 (PMC10091157; doi:10.1001/jamanetworkopen.2023.6951)
Supplement: Supplement 1. — eTable. Suicides Around the Anniversary of a Parent’s Death Among Individuals Aged 18-65 Who Experienced Parental Death Between 1991 and 2015 (n=7,694) eFigure 1. Flowchart of the Study Population eFigure 2. Example of Selection of Control Days, for Case Day on Monday, March 8, 2010 eFigure 3. Target Periods for Each Exposure Variable eFigure 4. Illustration of How the Dataset and Variables Were Created for the Case-Crossover Study eFigure 5. Association Between the Anniversary of a Parent’s Death and Suicide Among Women and Men, Stratified by Time Since Parental Death eFigure 6. Association Between the Anniversary of a Parent’s Death and Suicide Among Women and Men Using Semielasticities eFigure 7. Association Between the Anniversary of a Parent’s Death and Suicide Among Women and Men (Sensitivity Analysis After Randomly Selecting 1 Control Day) [file jamanetwopen-e236951-s001.pdf]

Supplemental Online Content

Grotta A, Liu C, Hiyoshi A, et al. Suicide around the anniversary of a parent’s death in Sweden. *JAMA Netw Open*. 2023;6(4):e236951. doi:10.1001/jamanetworkopen.2023.6951

eTable. Suicides Around the Anniversary of a Parent’s Death Among Individuals Aged 18-65 Who Experienced Parental Death Between 1991 and 2015 (n=7,694)

eFigure 1. Flowchart of the Study Population

eFigure 2. Example of Selection of Control Days, for Case Day on Monday, March 8, 2010

eFigure 3. Target Periods for Each Exposure Variable

eFigure 4. Illustration of How the Dataset and Variables Were Created for the Case-Crossover Study

eFigure 5. Association Between the Anniversary of a Parent’s Death and Suicide Among Women and Men, Stratified by Time Since Parental Death

eFigure 6. Association Between the Anniversary of a Parent’s Death and Suicide Among Women and Men Using Semielasticities

eFigure 7. Association Between the Anniversary of a Parent’s Death and Suicide Among Women and Men (Sensitivity Analysis After Randomly Selecting 1 Control Day)

This supplemental material has been provided by the authors to give readers additional information about their work.

© 2023 Grotta A et al. *JAMA Network Open.*

eTable. Suicides Around the Anniversary of a Parent's Death Among Individuals Aged 18-65 Who Experienced Parental Death Between 1991 and 2015 (n=7,694)

	Women (n=2,255)		Men (n=5,439)		Total (n=7,694)	
	**No.**	**%**	**No.**	**%**	**No.**	**%**
**Suicides occurring on the anniversary**	8	0.35	8	0.15	16	0.21
**Suicides occurring during 14 days before/after the anniversary**	169	7.49	398	7.32	567	7.37

© 2023 Grotta A et al. *JAMA Network Open.*

eFigure 1. Flowchart of the Study Population

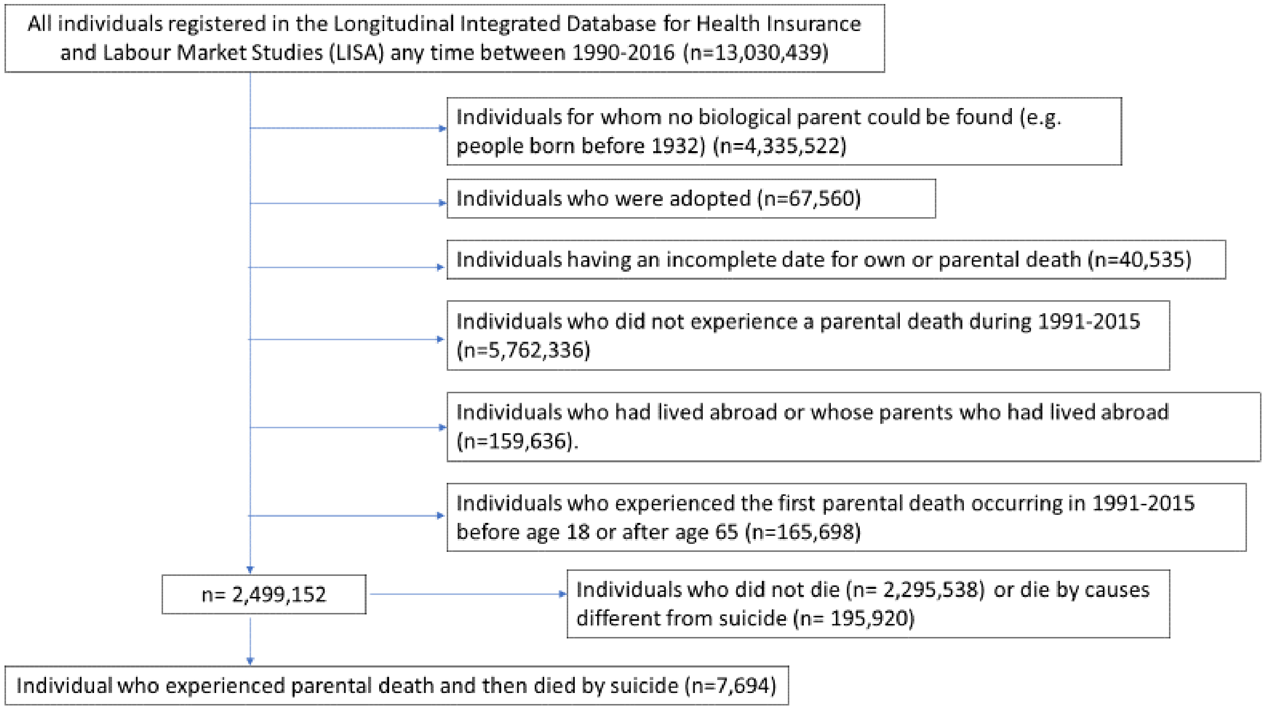

© 2023 Grotta A et al. *JAMA Network Open.*

eFigure 2. Example of Selection of Control Days, for Case Day on Monday, March 8, 2010

March 2010

Mon	Tue	Wed	Thu	Fri	Sat	Sun
1	2	3	4	5	6	7
8	9	10	11	12	13	14
15	16	17	18	19	20	21
22	23	24	25	26	27	28
29	30	31				

case day: the day of suicide of the offspring

control day: same days of the week within the same month of the same year

© 2023 Grotta A et al. *JAMA Network Open.*

eFigure 3. Target Periods for Each Exposure Variable

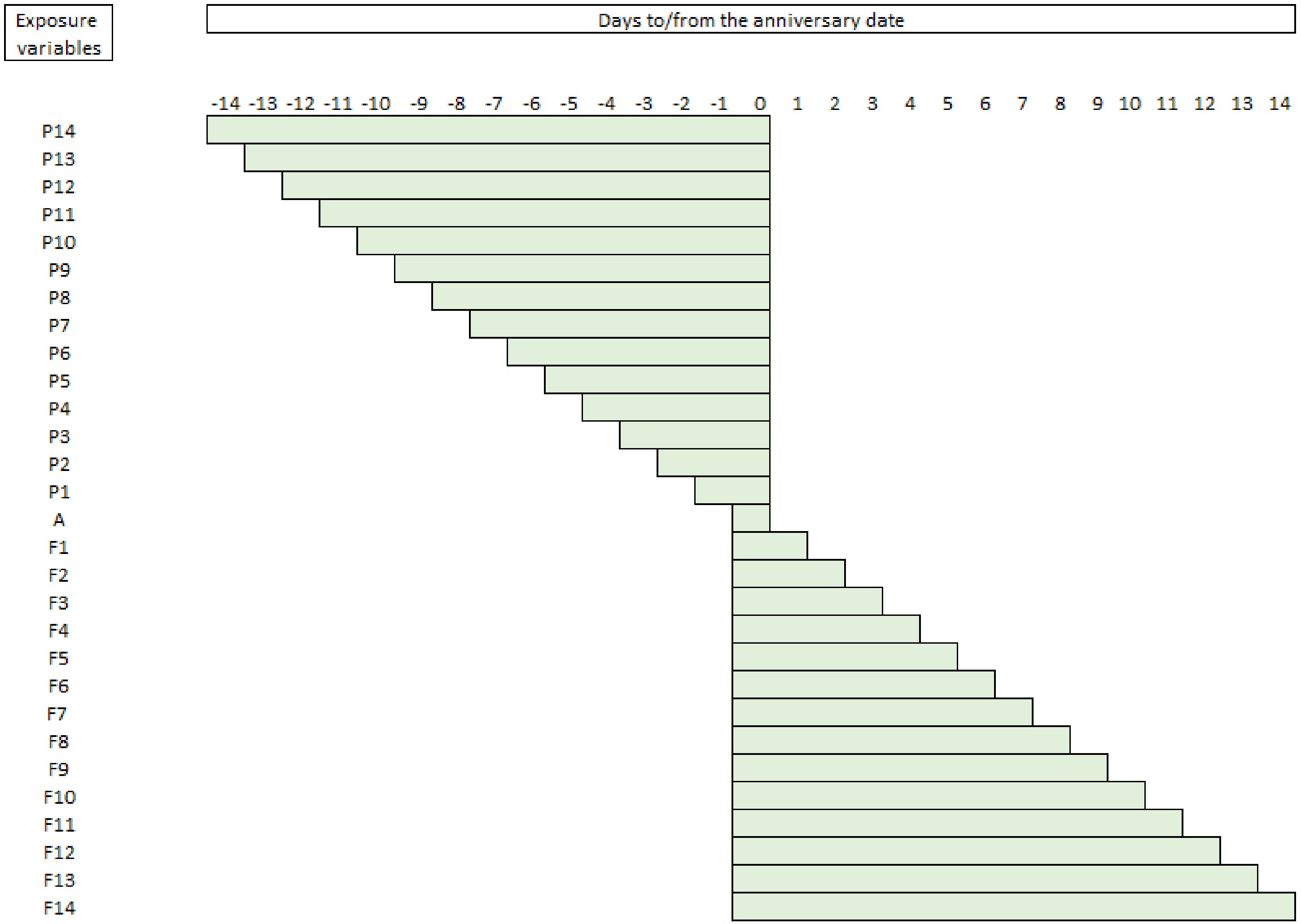

Pn: preceding the day of anniversary by n days
Fn: following the day of anniversary by n days

© 2023 Grotta A et al. *JAMA Network Open.*

eFigure 4. Illustration of How the Dataset and Variables Were Created for the Case-Crossover Study

ID	CASE/ CONTROL	CASE DAY	CONTROL DAY	ANNIVERSARY DAY	P14	P13	P12	P11	P10	P9	P8	P7	P6	P5	P4	P3	P2	P1	A	F1	F2	F3	F4	F5	F6	F7	F8	F9	F10	F11	F12	F13	F14
124	1	8 March 2010		3 March	0	0	0	0	0	0	0	0	0	0	0	0	0	0	0	0	0	0	0	1	1	1	1	1	1	1	1	1	1
124	0		1 March 2010	3 March	1	1	1	1	1	1	1	1	1	1	1	1	1	0	0	0	0	0	0	0	0	0	0	0	0	0	0	0	0
124	0		15 March 2010	3 March	0	0	0	0	0	0	0	0	0	0	0	0	0	0	0	0	0	0	0	0	0	0	0	0	0	0	1	1	1
124	0		22 March 2010	3 March	0	0	0	0	0	0	0	0	0	0	0	0	0	0	0	0	0	0	0	0	0	0	0	0	0	0	0	0	0
124	0		29 March 2010	3 March	0	0	0	0	0	0	0	0	0	0	0	0	0	0	0	0	0	0	0	0	0	0	0	0	0	0	0	0	0

ID: personal identifier

CASE/CONTROL: indicator variable for cases (1) and controls (0)

CASE DAY: day of the suicide

CONTROL DAY: same days of the week within the same month of the same year as for the case day

ANNIVERSARY DAY: anniversary of parental death

Explanations for variables from P14 - F14:

Pn: indicator variables taking values 1 if the case (or control) day falls in the time period ranging from n days preceding the anniversary to the anniversary

A: indicator variables taking values 1 if the case (or control) day overlaps with the anniversary

Fn: indicator variables taking values 1 if the case (or control) day falls in the time period ranging from the anniversary to n days following the anniversary

© 2023 Grotta A et al. *JAMA Network Open.*

eFigure 5. Association Between the Anniversary of a Parent's Death and Suicide Among Women and Men, Stratified by Time Since Parental Death

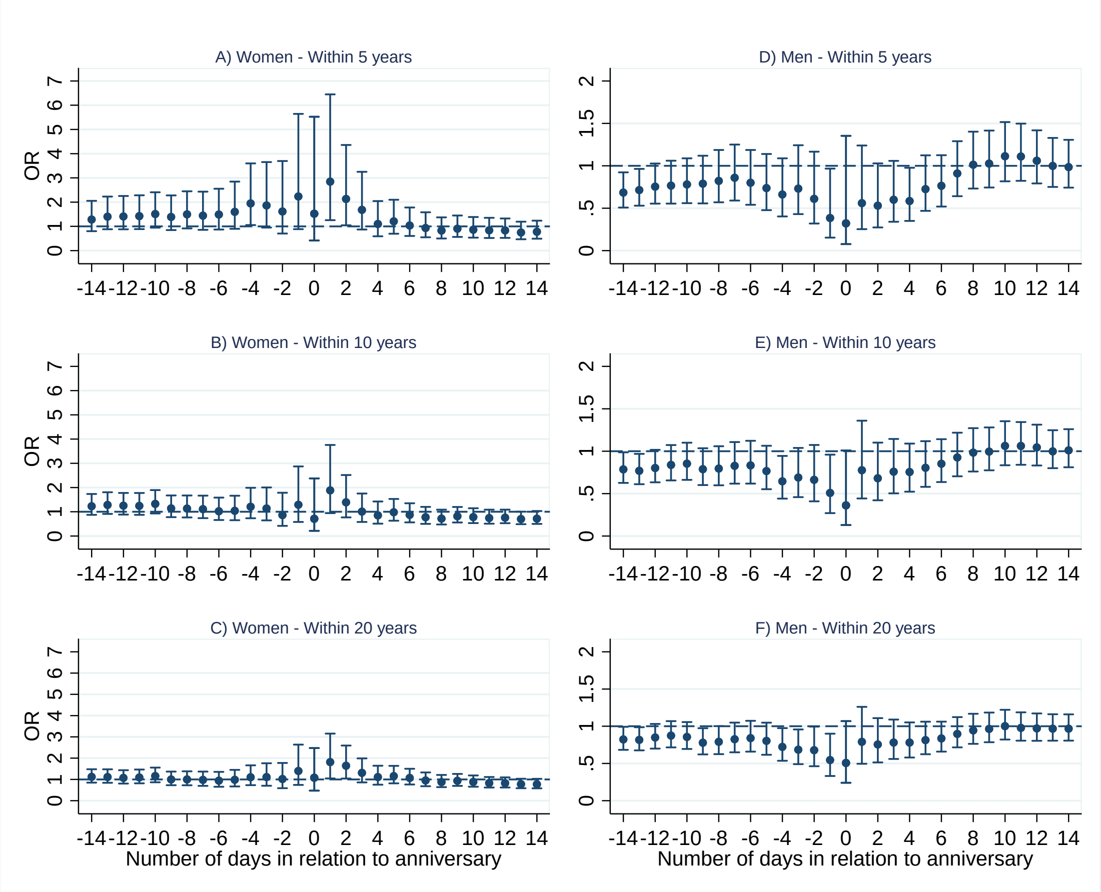

Odds ratios (OR) and 95% confidence intervals (95% CI) for the association between anniversary (or pre/post anniversary periods) and suicide among women restricting on suicides occurring within five (panel A and D), ten (panel B and E) and twenty (panel C and F) years following parental death.

© 2023 Grotta A et al. *JAMA Network Open.*

eFigure 6. Association Between the Anniversary of a Parent's Death and Suicide Among Women and Men Using Semielasticities

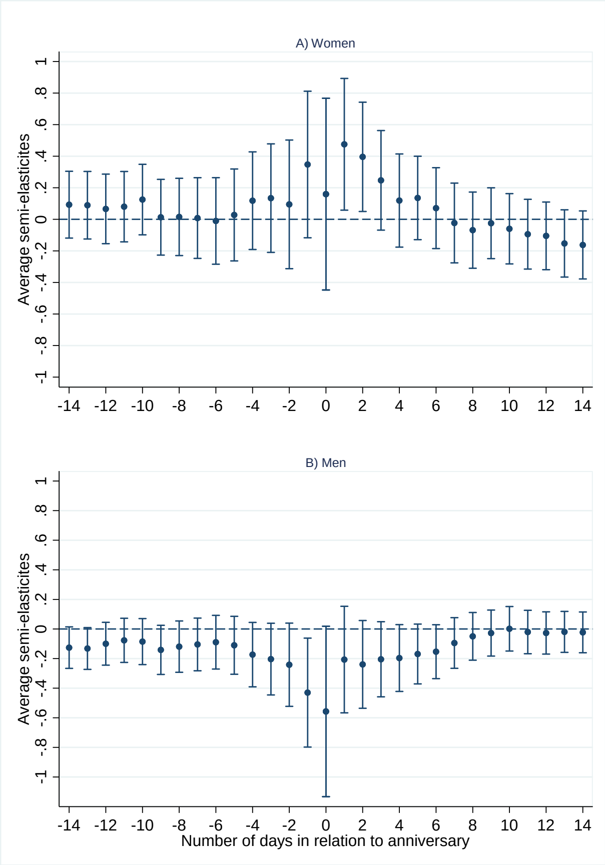

Semielasticities and 95% confidence intervals (95% CI) for the association between anniversary (or pre/post anniversary periods) and suicide among women (panel A) and men (panel B)

© 2023 Grotta A et al. *JAMA Network Open.*

eFigure 7. Association Between the Anniversary of a Parent's Death and Suicide Among Women and Men (Sensitivity Analysis After Randomly Selecting 1 Control Day)

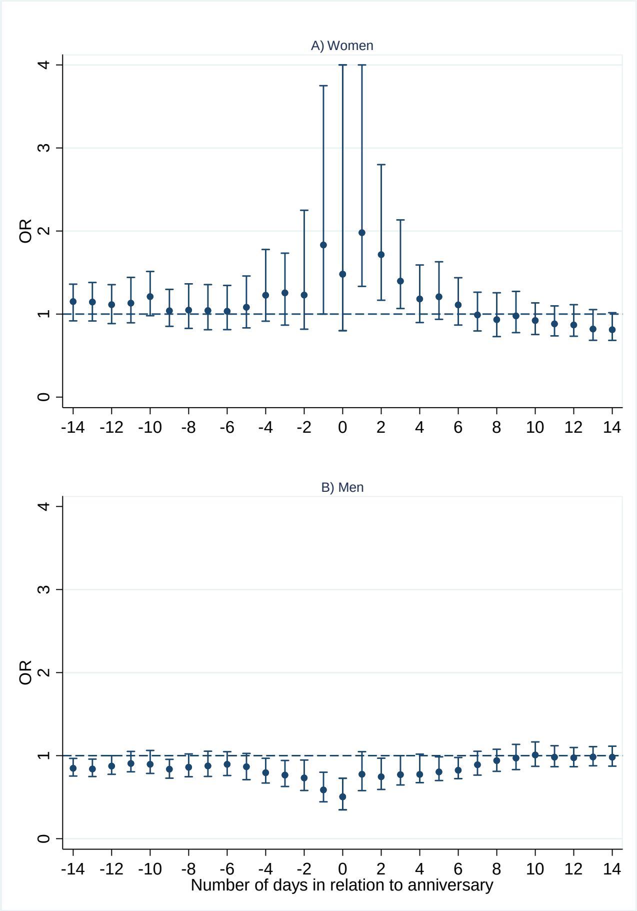

Odds ratios (OR) and 95% confidence intervals (95% CI) for the association between anniversary (or pre/post anniversary periods) and suicide among women (panel A) and men (panel B): sensitivity analysis after randomly selecting one control day. We repeated the sampling process of the control day 100 times and plotted the average OR with the 2.5th and 97.5th percentiles.

© 2023 Grotta A et al. *JAMA Network Open.*
