# Supplementary material for: Suicide Around the Anniversary of a Parent’s Death in Sweden
Source: JAMA Netw Open. 2023 Apr 11;6(4):e236951. doi: 10.1001/jamanetworkopen.2023.6951 (PMC10091157; doi:10.1001/jamanetworkopen.2023.6951)
Supplement: Supplement 2. — Data Sharing Statement [file jamanetwopen-e236951-s002.pdf]

Data Sharing Statement

Grotta. Suicide Around the Anniversary of a Parent's Death in Sweden. *JAMA Netw Open*. Published April 11, 2023. doi:10.1001/jamanetworkopen.2023.6951

Data
Data available: No

Additional Information
Explanation for why data not available: The source datasets supporting the conclusions of this article are available in the Swedish agencies´ repositories and can be made available to researchers in accordance with the ethical and legal restrictions regarding Swedish Public Access to Information and Secrecy Act data.
